# *CDKN1C*/p57^kip2 ^is a candidate tumor suppressor gene in human breast cancer

**DOI:** 10.1186/1471-2407-8-68

**Published:** 2008-03-06

**Authors:** Pamela S Larson, Benjamin L Schlechter, Chia-Lin King, Qiong Yang, Chelsea N Glass, Charline Mack, Robert Pistey, Antonio de las Morenas, Carol L Rosenberg

**Affiliations:** 1 Department of Pathology and Laboratory Medicine, Boston University Medical Center, Boston MA USA; 2 Department of Medicine, Boston University Medical Center, Boston MA, USA; 3 Department of Biostatistics, Boston University School of Public Health, Boston MA, USA; 4 Division of Graduate Medical Sciences, Boston University Medical Sciences, Boston MA, USA

## Abstract

**Background:**

CDKN1C (also known as p57^KIP2^) is a cyclin-dependent kinase inhibitor previously implicated in several types of human cancer. Its family members (CDKN1A/p21^CIP1 ^and B/p27^KIP1^) have been implicated in breast cancer, but information about CDKN1C's role is limited. We hypothesized that decreased CDKN1C may be involved in human breast carcinogenesis *in vivo*.

**Methods:**

We determined rates of allele imbalance or loss of heterozygosity (AI/LOH) in *CDKN1C*, using an intronic polymorphism, and in the surrounding 11p15.5 region in 82 breast cancers. We examined the CDKN1C mRNA level in 10 cancers using quantitative real-time PCR (qPCR), and the CDKN1C protein level in 20 cancers using immunohistochemistry (IHC). All samples were obtained using laser microdissection. Data were analyzed using standard statistical tests.

**Results:**

AI/LOH at 11p15.5 occurred in 28/73 (38%) informative cancers, but *CDKN1C *itself underwent AI/LOH in only 3/16 (19%) cancers (p = ns). In contrast, CDKN1C mRNA levels were reduced in 9/10 (90%) cancers (p < 0.0001), ranging from 2–60% of paired normal epithelium. Similarly, CDKN1C protein staining was seen in 19/20 (95%) cases' normal epithelium but in only 7/14 (50%) cases' CIS (p < 0.004) and 5/18 (28%) cases' IC (p < 0.00003). The reduction appears primarily due to loss of CDKN1C expression from myoepithelial layer cells, which stained intensely in 17/20 (85%) normal lobules, but in 0/14 (0%) CIS (p < 0.00001). In contrast, luminal cells displayed less intense, focal staining fairly consistently across histologies. Decreased CDKN1C was not clearly associated with tumor grade, histology, ER, PR or HER2 status.

**Conclusion:**

CDKN1C is expressed in normal epithelium of most breast cancer cases, mainly in the myothepithelial layer. This expression decreases, at both the mRNA and protein level, in the large majority of breast cancers, and does not appear to be mediated by AI/LOH at the gene. Thus, *CDKN1C *may be a breast cancer tumor suppressor.

## Background

Cyclin-dependent kinases (CDK) are a family of enzymes that govern the mammalian cell cycle (among other functions) and whose aberrant upregulation can lead to oncogenic effects. Consequently, proteins that negatively regulate CDKs, cyclin-dependent kinase inhibitors (CDKNs), are strong candidate tumor suppressor genes. CDKNs fall into two families. The CDKN1 family contains three members, called CDKN1A (also known as p21^CIP^) [1], CDKN1B (also known as p27^KIP1^) [2,3] and CDKN1C (also known as p57^KIP2^) [4,5]. CDKN1A and B play important and complex roles in breast cancer, but knowledge of CDKN1C's role is limited. The goal of this study is to examine whether CDKN1C is implicated in human breast cancers *in vivo*.

CDKN1C is a tight-binding inhibitor of several G1 cyclin/CDK complexes and a negative regulator of the cell cycle at the G1 checkpoint (for overview see [6]). The protein is widely expressed and located in the nucleus. The gene lies within an imprinted region, at chromosome 11p15.5-p15.4, and undergoes incomplete paternal imprinting, i.e., the maternal allele is expressed preferentially [7]. *CDKN1C *dysregulation – usually assessed as gene methylation or decreased mRNA expression – is seen in multiple types of sporadic cancers (adrenal [8,9], head and neck [10,11], gastrointestinal [12-15], urothelial [16,17] and lung carcinomas [18-20]), as well as in gestational trophoblastic disease [21], Wilms' tumor [22] and the Beckwith-Wiedemann syndrome associated with organ overgrowth [23]. Over-expression, or re-expression, of the gene *in vitro *slows proliferation and shifts many cell types into G1 [24-26].

Several *in vitro *observations support the hypothesis that CDKN1C is implicated in breast tumorigenesis. First, decreased CDKN1C expression occurs during human mammary epithelial cell immortalization [27]. Second, CDKN1C may be regulated by estradiol in mammary carcinoma cells [28]. Finally, CDKN1C may be regulated by epigallocatechin-3-gallate (EGCG), a polyphenol in green tea with anti-oxidant effects that may have cancer-suppressive effects in general, and in breast cancer in particular [29-32].

CDKN1C's role in breast cancer *in vivo *has been considered previously, but data are limited. 11p15 undergoes allele imbalance (AI) or loss of heterozygosity (LOH) in 35–40% of breast cancers [33-35], but CDKN1C does not appear to be commonly mutated or rearranged [34,36]. The gene is hypermethylated in ~45% of primary tumors [18]. Gene expression data available in repositories [37] reveals that CDKN1C mRNA is present at relatively low levels in both normal and cancerous breast tissue. We are not aware of any reports examining CDKN1C protein expression in human breast cancers.

Taken together, these data led us to investigate CDKN1C's potential role as a tumor suppressor in breast cancer *in vivo*. We speculated that the gene itself would not undergo genomic alterations detectable by AI/LOH, but that CDKN1C mRNA and protein levels would be diminished. To ascertain whether the genetic alterations were present in the epithelial compartment, as presumed, all tissues examined were microdissected.

## Methods

### Tissue samples

After obtaining Boston University Medical Center institutional review board approval, randomly-selected, de-identified, existing tissue samples not needed for diagnosis were collected from 91 independent breast cancer cases from Boston University Medical Center. In 87 cases, tissues had been formalin-fixed and paraffin embedded (FFPE). In 10 cases, tissues had been snap frozen, embedded in optimal cutting temperature (OCT) medium and stored at -80°C. One or more experienced breast pathologist (AdlM, RP) reviewed all sections for accurate histologic diagnoses.

### Microdissection, DNA isolation and AI/LOH analysis

These procedures were carried out as described previously [38-40]. FFPE or OCT embedded blocks were sectioned and microdissected (PixCell, Arcturus Engineering, Mountain View, CA) to isolate DNA from normal epithelium, tumor and control tissue. PCRs were performed using nine microsatellite probes: four at 11p15.4-.5 (D11s2071, D11s1318, THO1 and KIP2 – which is located in a *CDKN1C *intron); 3 at 11q (PYGM (at 11q13.1) and D11s1818 and D11s1819 (at 11q23.1-.2), and one each at 3p24.2-5 (D3s1283) and 7q31 (D7s486), which served as control loci. Either a radioactive or fluorescent [41] label was incorporated into the reactions. The normal pattern at each microsatellite probe was defined as its pattern in normal tissue of each individual (skin, lymph node or normal breast epithelium). AI/LOH was defined as an imbalance of 33% or more (allele ratio >1.50 or <0.67). All abnormalities were demonstrated at least twice with equivalent results.

### Microdissection, RNA isolation and qPCR

Cases with available tissue were selected at random and their OCT-embedded blocks were sectioned and microdissected with the aid of pathologists to isolate normal breast epithelium (terminal ducto-lobular units, or TDLUs) and tumor [42]. Control RNA was extracted (Qiagen RNeasy Midi Kit, Qiagen, Valencia CA) from bulk normal-appearing tissue from a cancer-containing breast stored in RNAlater (Qiagen). To prevent clogging of the RNeasy column by excess fat, the tissue homogenate was spun at 6100 rpm for 10 minutes at 4°C and only the aqueous layer was used for RNA purification. All RNA samples were quantified spectrophotometrically (Nanodrop Technologies, Wilmington, DE), and 1.0 ng RNA from each sample was run on an RNA 6000 Pico LabChip for the Agilent Bioanalyzer 2100 (Agilent Technologies, Waldbronn, Germany) to confirm quality.

To produce cDNA, RT-PCR was performed on 5.0 ng of each microdissected RNA sample using Taqman Gold RT-PCR reagents (Applied Biosystems, Foster City, CA) in a 25 μl reaction. Quantitative real-time PCR (qPCR) was performed using Taqman Universal PCR Mastermix (ABI), in independent 25 μl reactions consisting of 11.25 μl cDNA solution and an Assay on Demand (ABI) for *CDKN1C *or glucuronidase *B *(*GUSB)*, an endogenous control gene located at 7q31 whose expression is similar in normal and malignant breast tissue [43]. The *CDKN1C *primers are designed to amplify the 3' end of the first and the 5' end of the second exon. QPCR was performed using an ABI Prism 7000 Sequence Detection System and analyzed using the SDS version 1.1 software (ABI). Standard curves using the control RNA isolated from bulk normal breast tissue were performed with every reaction. All reactions were done twice, and an independent RT-PCR and qPCR was performed for each replicate to reduce error and allow greater accuracy when comparing reactions done at different times.

### Immunohistochemistry (IHC)

Cases with available FFPE blocks were selected at random and 5 μm sections were cut from FFPE blocks, mounted on positively charged glass slides, dried at 60°C for 1 hr, and deparaffinized. Endogenous peroxidase activity was quenched with hydrogen peroxide/methanol for 15 mins. Antigen retrieval was achieved by microwaving the slides in a citrate buffer (Citrate Plus, Biogenex, San Ramon, CA) on the high setting for 3–5 mins followed by the medium setting for 8 mins. After washing, sections were incubated for 1 hr at room temperature with a rabbit polyclonal anti-CDKN1C antibody directed against the carboxy-terminus of the protein (C-20, Santa Cruz, CA) at a 1:2000 dilution of the stock solution. The negative control was a rabbit polyclonal antibody pool (Biogenex). To localize sites of primary antibody binding, sections were incubated with a biotin-labeled goat anti-rabbit secondary antibody (20 mins) followed by streptavidin-HRP (20 mins) (LP000-UL, Biogenex). After washing, sections were exposed to the chromagen diaminobenzidine for 10 sec. before counterstaining with hematoxylin for 30 sec.

Two pathologists [RP, AdlM] independently reviewed the slides and recorded the proportion of cells with nuclear staining in each type of lesion (normal epithelium, CIS, IC). The proportion of cells with staining was assessed separately for the myoepithelial and luminal regions. The intensity of staining was graded using a semi-quantitative 0–3^+ ^scale (0 = no staining, 1^+ ^= weak, 2^+ ^= moderate, 3^+ ^= strong). Placenta served as the positive control.

### Statistical analyses

To compare rates of AI/LOH at separate probes, Fisher's exact test was used. For the RNA expression data, Friedman's two-way non-parametric analysis of variance (ANOVA) was used to compare two matched groups (normal and tumor) and each group was measured two times. Within each sample, the values of the difference between the Ct values (ΔCt) of *CDKN1C *and *GUSB *were ranked. Then the ranks were compared between the normal and tumor groups to form a chi-square statistic with one degree of freedom. For the IHC data, Fisher's exact test was used to test the difference between two proportions (normal and CIS, normal and IC, or CIS and IC).

## Results

### Cases

Ninety-one breast cancers were analyzed. Of these, DNA from 82 were analyzed for AI/LOH at four 11p15.5 and five control loci, RNA from ten cases were analyzed for expression of CDKN1C mRNA via qPCR, and tissues from 20 cases were analyzed for staining of CDKN1C protein via IHC. The clinical-pathologic characteristics of the cases used suggest that they represent an unselected group of breast cancers. These characteristics are summarized in Table 1.

**Table 1:** Clinico-pathologic characteristics of study cases

	DNA cases *(%)*	RNA cases *(%)*	Protein cases *(%)*

Total number of cases*	82	10	20
Age (years)	50	53	48
Age range (years)	33 to 92	33 to 92	30 to 62
Ductal carcinoma	76/81 *(94)*	10/10 *(100)*	20/20 *(100)*
Lobular carcinoma	6/81 *(6)*	0/10 *(0)*	0/20 *(0)*
Grade			
1 of 3	5/71 *(7)*	0/10 *(0)*	1/20 *(5)*
2 of 3	18/71 *(25)*	3/10 *(30)*	6/20 *(30)*
3 of 3	48/71 *(68)*	7/10 *(70)*	13/20 *(65)*
ER and/or PR positive	45/65 *(69)*	7/10 *(70)*	15/20 *(75)*
Her2/neu positive	8/36 *(22)*	2/10 *(20)*	3/20 *(15)*

* In some cases complete clinico-pathologic data were not available

### DNA

82 breast cancer cases were analyzed for AI/LOH (nine CIS, 73 IC). The results are summarized in Table 2. AI/LOH at one or more markers on 11p15 was seen in 28/73 (38%) informative tumors, consistent with previous reports [33,34,44-46]. In contrast, AI/LOH at *CDKN1C *itself was seen in 3/16 (19%) informative tumors, a level consistent with a background or non-specific rate. Results were the same from frozen and FFPE samples. There were no statistical differences between the rates of AI/LOH at *CDKN1C *and any other 11p15 probe.

**Table 2:** No increase in AL/LOH at *CDKN1C *in 82 primary breast cancers

Chromosomal locus and probes	Number of cancers with AI/LOH *(%)*
11p15.4-.5	28/73 *(38)*
D11s2071	20/45 (*44*)
THO1	19/57 (*33*)
D11s1318	06/15 (*40*)
*CDKN1C *(KIP2)	03/16 (*19*)
11q13.1	09/32 *(29*)
PYGM	09/32 *(29)*
11q23.1-.2	34/71 (*48*)
D11s1818	24/55 (44)
D11s1819	23/47 (49)
3p24.2-5	09/27 (*33*)
D3s1283	09/27 (*33*)
7q31	08/39 (21)
D7s486	08/39 (21)

### RNA

**Figure 1:**
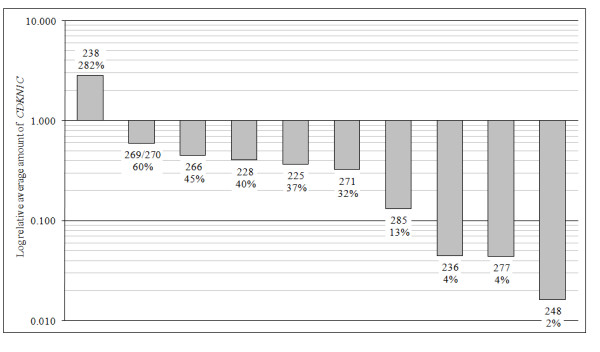
***CDKN1C *mRNA levels are decreased in primary breast cancers**. Expression of *CDKN1C *mRNA in cancers and paired normal epithelium were measured by qPCR, and each was normalized against an endogenous control, *GUSB*. RNA levels are displayed on a logarithmic scale. Each bar represents the average results for each case. Bars are labeled with the case number and the percent level of expression of *CDKN1C *in the tumor compared to its own normal.

Cancer and paired normal epithelium from ten invasive breast cancers were analyzed for CDKN1C RNA expression by qPCR. We found reduced expression of CDKN1C in 9/10 (90%) cancers relative to its paired normal epithelium, and normalized against *GUSB *(see Figure 1). The ΔCt between *CDKN1C *and *GUSB *for each normal sample was compared to its paired tumor sample to generate the relative expression of CDKN1C mRNA between tumor and normal. This difference in relative expression of mRNA between the cancer and normal groups was significant (p < 0.0001). The fold changes in mRNA level were moderate. As shown in Figure 1, CDKN1C mRNA levels in nine cancers ranged from 2 – 60% of normal and in one cancer was 282% of normal.

### Protein

**Figure 2:**
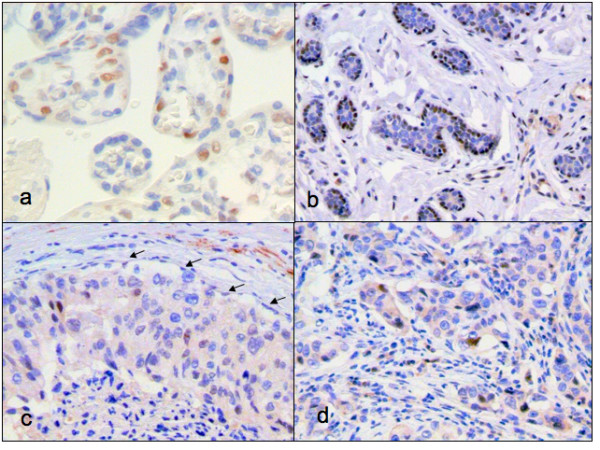
**Representative examples of CDKN1C immunohistochemistry**. a) placenta, (positive control) with 3+ nuclear staining; b) normal breast epithelium, with 3+ nuclear staining located in the area of myoepithelium and no staining in the luminal epithelium; c) DCIS, with no staining in the myoepithelium (see arrows) and 1–2+ focal nuclear staining in the malignant luminal epithelium; d) IDC, with 1–2+ focal nuclear staining in malignant epithelium. 200×.

**Figure 3:**
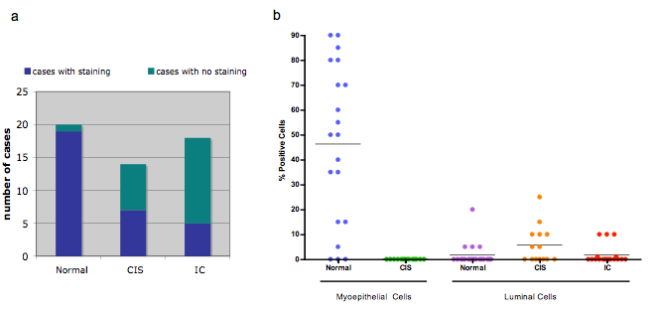
**CDKN1C protein is decreased in breast cancer**. a) The number of cases with positive staining for CDKN1C is plotted for each tissue type (normal epithelium, CIS and IC). b) The percent of cells staining for CDKN1C is plotted for myoepithelial and luminal area cells, in normal epithelium, CIS and IC. Each dot represents a separate case and the horizontal line indicates the median. IC by definition contain no myoepithelial cells.

Twenty breast cancer cases were examined for CDKN1C protein levels using IHC. Figure 2 shows representative examples of staining. Overall, there was less CDKN1C staining in both CIS and IC, compared to normal epithelium. As shown in Figure 3a, staining of any intensity at any location was present in 19/20 (95%) normal epithelial samples, compared to 7/14 (50%) CIS (p = 0.004) and 5/18 (28%) IC (p = 0.00002). The difference between CIS and IC was not significant.

CDKN1C protein expression did not appear to be distributed uniformly between the luminal and myoepithelial layers. In normal lobules, the positively staining cells were predominantly in the myoepithelial layer, as seen in Figure 2b. These cells stained intensely, usually 2+ – 3+. On average, 46% of myoepithelial layer cells, from 17/20 (85%) cases, stained for CDKN1C. In contrast, no myoepithelial layer cells (0%) stained for CDKN1C in any CIS (0/14 (0%) cases). An example is shown in Figure 2c. By definition, IC does not have a myoepithelial layer. In luminal cells, CDKN1C staining was generally more focal, less common, and less intense (1+ – 2+) than in myoepithelial layer cells. In addition, luminal cell CDKN1C staining was fairly similar across the three histologies. In normal lobules, luminal cell staining was confined to 2% of cells, from 4/20 (20%) cases, in CIS it was limited to 6% of cells, from 7/14 (50%) cases, and IC it was seen in 2% of cells from 5/18 (28%) cases. These results are depicted in Figure 3b. Table 3 presents the IHC results for each case.

**Table 3:** CDKN1C protein expression by IHC in 20 breast cancer cases

Case	225	243	248	261	266	267	271	273	277	285	7018	7028	7030	7031	7036	7037	7040	7042	7043	7049
Myoepithelium (% of cells staining positive for CDKN1C)																				
TDLU	0	35	0	5	85	40	70	80	35	15	50	55	50	15	0	90	90	70	80	60
CIS	0	0	0	0		0	0	0		0		0		0			0	0	0	0
IC*																				
Luminal epithelium (% of cells staining positive for CDKN1C)																				
TDLU	20	0	5	0	0	0	0	0	0	0	0	0	0	0	0	0	0	0	5	5
CIS	25	0	0	0		10	0	5		0		0		10			15	0	5	10
IC		0	0	0	0		0	10	0	0	0	0	0	10	0	1	0	0	1	10

*By definition the invasive component does not have a myoepithelial layer. Blank box, not available for analysis

### Integration of DNA, RNA, and protein data

There were six cases with DNA, mRNA and protein data. The findings in these cases were consistent with those of the full group. None of the six had AI/LOH at *CDKN1C*, but all had decreased mRNA levels. CDKN1C protein staining was present in normal epithelium of 6/6 (100%) cases (4 in the myoepithelial and 2 in luminal area cells), and complete loss of staining was seen in 3/4 (75%) CIS and 5/5 (100%) IC. These data are summarized in Table 4.

**Table 4:** Clinico-pathologic, DNA, RNA, and protein analysis of CDKN1C in six cases with breast cancer

Case number	225	248	266	271	277	285
Age (years)	40	48	46	39	56	50
Histology	D	D	D	D	D	D
Grade (out of 3)	3	2	3	3	3	2
ER/PR	+/+	+/+	+/+	+/+	-/-	+/+
Her2/neu	+	-	-	-	-	-
						
DNA – analysis of AI/LOH						
11p15.4-.5	-	-	-	-	+	
*CDKN1C*	-		-	-		
						
mRNA – % of normal *CDKN1C *expression						
	37	2	45	32	4	13
						
Protein – staining for CDKN1C						
Myoepithelium						
Normal	-	-	+	+	+	+
CIS	-	-		-		-
IC*						
						
Luminal epithelium						
Normal	+	+/-	-	-	-	-
CIS	+	-		-		-
IC		-	-	-	-	-

D, ductal histology; blank box, not available.

+, positive staining, amplification or AI/LOH;

-, negative staining, amplification or no AI/LOH.

CIS, carcinoma *in situ*; IC, invasive carcinoma.

*By definition invasive carcinoma does not have a myoepithelium

### Clinical-pathological correlation

We tested for associations between AI/LOH at each DNA marker,*CDKN1C *mRNA expression and protein staining, and subject age, tumor histology, grade, ER/PR or HER2 status. The only association seen was between AI/LOH at the *THO1 *marker and lack of ER/PR expression (p = 0.036). The other chromosome 11p markers, including *CDKN1C*, were not significantly associated, nor was AI/LOH at THO1 associated with other clinical-pathological features.

## Discussion

We speculated that CDKN1C might, like its family members, be implicated in breast carcinogenesis *in vivo*. Therefore, we investigated its DNA, mRNA and protein levels in a series of primary breast cancers, compared to paired normal breast epithelium. Using microdissected samples, we found that the gene does not appear to undergo AI/LOH, but its mRNA level is reduced in 90% of cancers, and its protein is completely absent in 50% of *in situ *and 72% of invasive cancers. The reduction appears primarily due to loss of CDKN1C expression from myoepithelial layer cells, which stained intensely in 17/20 (85%) normal lobules, but in 0/14 (0%) CIS (p < 0.00001). AI/LOH of the 11p15.4-5 region in general and decreased expression of CDKN1C was not clearly associated with tumor grade, histology, ER, PR or HER2 status. These data suggest CDKN1C acts as a tumor suppressor in breast cancer, perhaps in myoepithelial cells, and that its function may be lost at or before the appearance of CIS. This study does not investigate the mechanism of the gene's inactivation, but our results are consistent with an epigenetic process, or RNA interference, although other processes could be involved as well.

These data raise several points for consideration. Gene expression profiling data available at Gene Expression Omnibus (last accessed 01-10-08 [37]) indicate that CDKN1C mRNA expression is relatively low and not consistently different between normal and cancerous breast tissue. The data from primary tissues generally derive from analysis of bulk normal and tumor samples that contain a heterogeneous mix of cells. In contrast, we saw small but widespread decreases in *CDKN1C *mRNA expression in malignant epithelium, compared to normal. We may have observed subtle but consistent decreases because we microdissected our samples, which enriched them for epithelium and unmasked the gene's potentially important role. This speculation is supported by our finding that in normal epithelium the expression appears localized mainly in myoepithelium, which constitutes only a fraction of the cells of the normal lobule. It is believed that a functioning myoepithelium may suppress progression of the neoplastic luminal cells [47]. Loss of myoepithelial CDKN1C protein expression could participate in the dysregulation of this process, resulting in cancer progression. We note, however, that CDKN1C mRNA was not found to be differentially expressed in one study comparing myoepithelial with luminal cells [48]. This may reflect the techniques used, or effects of post-transcriptional modifications leading to lack of concordance between mRNA and protein. Furthermore, we cannot distinguish whether the decreased CDKN1C mRNA expression we observe in microdissected epithelium is due to all cells having moderately decreased expression, or to a subpopulation [e.g., myoepithelial cells] having dramatically decreased expression, or to a decrease in the number of cells [e.g., myoepithelial cells] with constant high expression. Future experiments to address this question will be important.

Secondly, we see decreased mRNA in nearly all cases, but the gene is reported to be methylated in only half that number [18]. One potential explanation for this discrepancy is that gene methylation may really be present in nearly all cases, but has been hidden due to "contamination" by heterogeneous non-malignant cells with an unmethylated gene. Another possibility is that alternative mechanisms, such as histone modification, or post-transcriptional effects, may lead to decreased RNA and, subsequently, protein, levels. Decreased CDKN1C mRNA expression due to several mechanisms has recently been described in primary pancreatic neoplasms [15].

Third, it is unknown how CDKN1C interacts with its family members. The genes probably have some functional redundancy, but there could be subsets of cells within breast tissue with restricted expression of one or another CDKN1 family member [49]. The CDKN1 family may parallel the CDKN2 family, which also contains several members with clear roles in carcinogenesis and one member, (CDKN2B, or p15), whose role is unresolved.

There are several potential limitations to the present study. First, our evaluation of DNA alterations is not comprehensive. We opted for this approach because previous studies found sequence alterations to be uncommon, and gene methylation to be present in nearly half of breast cancers. Second, the number of cases examined is relatively small. However, the results are remarkably uniform. Third, we used a single set of primers and a single antibody to examine RNA and protein levels, respectively, and thus may not have detected uncharacterized splice variants or aberrant proteins. *CDKN1C *contains 3 exons; the second and third are coding and produce two isoforms by alternative splicing. The long 316 amino acid (aa) isoform is unprocessed, and the short, 305 aa isoform is missing the first, or amino-terminal, 11 aa. Since we used an antibody directed at a carboxy-terminal epitope, both isoforms should have been detected. The RNA primers spanned the first and second exon and should have detected all transcripts. Fourth, we defined myoepithelial cells by histologic appearance and location, not by additional stains. Finally, this study found a strong association between CDKN1C and breast cancer, but cannot determine causality.

## Conclusion

We find that the *CDKN1C *gene does not appear to undergo frequent genetic alteration, as measured by AI/LOH, but that mRNA and protein levels are decreased in the large majority of breast cancers compared to paired normal epithelium. The reduction in protein expression appears mainly due to loss of expression in normal lobule's myoepithelial layer cells. Combining these data with existing knowledge of the gene's regulation and the protein's function, suggest that CDKN1C is a tumor suppressor that could act early in breast carcinogenesis, perhaps in the myoepithelial compartment.

## Abbreviations

aa: amino acid; AI/LOH: allele imbalance or loss of heterozygosity; CDKN1A cyclin-dependent kinase inhibitor family 1 member A (also known as p21); CDKN1B cyclin-dependent kinase inhibitor family 1 member B (also known as p27); CDKN1C cyclin-dependent kinase inhibitor family 1 member C (also known as p57); CDKN2 cyclin-dependent kinase inhibitor family 2; CIS: carcinoma *in situ*; EGCG: epigallocatechin-3-gallate; ER: estrogen receptor; HER2: human epidermal growth factor receptor 2; IC: invasive carcinoma; IHC: immunohistochemistry; PCR: polymerase chain reaction; PR: progesterone receptor

## Competing interests

The author(s) declare that they have no competing interests.

## Authors' contributions

PSL helped conceive, design and coordinate the study and carried out microdissections, DNA experiments, data analysis and helped write the paper; BLS also helped conceive and design the study and carried out DNA experiments, RNA experiments, data analysis and helped write the paper, CLK carried out microdissection, RNA experiments and data analysis, QY carried out statistical analyses, CG, CM, RP and AM performed immunohistochemistry, AM also contributed to data analysis, CLR helped conceive, design and coordinate the study, participated in the data analysis, and wrote the paper. All authors read and approved the final manuscript.

## Pre-publication history

The pre-publication history for this paper can be accessed here:



## References

[B1] Harper JW, Adami GR, Wei N, Keyomarsi K, Elledge SJ (1993). The p21 Cdk-interacting protein Cip1 is a potent inhibitor of G1 cyclin-dependent kinases. Cell.

[B2] Toyoshima H, Hunter T (1994). p27, a novel inhibitor of G1 cyclin-Cdk protein kinase activity, is related to p21. Cell.

[B3] Polyak K, Lee MH, Erdjument-Bromage H, Koff A, Roberts JM, Tempst P, Massague J (1994). Cloning of p27Kip1, a cyclin-dependent kinase inhibitor and a potential mediator of extracellular antimitogenic signals. Cell.

[B4] Lee M-H, Reynisdottir I, Massague J (1995). Cloning of p57KIP2, a cyclin-dependent kinase inhibitor with unique domain structure and tissue distribution. Genes & Development.

[B5] Matsuoka S, Edwards MC, Bai C, Parker S, Zhang P, Baldini A, Harper JW, Elledge SJ (1995). p57KIP2, a structurally distinct member of the p21CIP1Cdk inhibitor family, is a candidate tumor suppressor gene. Genes & Development.

[B6] De Clercq A, Inze D (2006). Cyclin-dependent kinase inhibitors in yeast, animals, and plants: a functional comparison. Critical reviews in biochemistry and molecular biology.

[B7] Matsuoka S, Thompson JS, Edwards MC, Barletta JM, Grundy P, Kalikin LM, Harper JW, Elledge SJ, Feinberg AP (1996). Imprinting of the gene encoding a human cyclin-dependent kinase inhibitor, p57KIP2, on chromosome 11p15. Proc Natl Acad Sci USA.

[B8] Liu J, Kahri AI, Heikkila P, Voutilainen R (1997). Ribonucleic acid expression of the clustered imprinted genes, p57KIP2, insulin-like growth factor II, and H19 in adrenal tumors and cultured adrenal cells. Journal of Clinical Endocrinology and Metabolism.

[B9] Bourcigaux N, Gaston V, Logic A, Bertagna X, Le Bouc Y, Gicquel C (2000). High expression of cyclin E and G1 CDK and loss of function of p57KIP2 are involved in proliferation of malignant sporadic adrenocortical tumors. J Clin Endocrinol Metab.

[B10] Fan GK, Chen J, Ping F, Geng Y (2006). Immunohistochemical analysis of P57(kip2), p53 and hsp60 expressions in premalignant and malignant oral tissues. Oral oncology.

[B11] Fan GK, Xu F, Yang B, Fujieda S (2006). p57(kip2) expression is related to carcinogenesis and tumor progression in laryngeal tissues. Acta oto-laryngologica.

[B12] Bonilla F, Orlow I, Cordon-Cardo C (1998). Mutational study of p16CDKN2/MTS1/INK4A and p57KIP2 genes in hetaptocellular carcinoma. Int J Oncol.

[B13] Ito Y, Takeda T, Sakon M, Tsujimoto M, Monden M, Matsuura N (2001). Expression of p57/Kip2 protein in hepatocellular carcinoma. Oncology.

[B14] Ito Y, Takeda T, Wakasa K, Tsujimoto M, Matsuura N (2001). Expression of p57/Kip2 protein in pancreatic adenocarcinoma. Pancreas.

[B15] Sato N, Matsubayashi H, Abe T, Fukushima N, Goggins M (2005). Epigenetic down-regulation of CDKN1C/p57KIP2 in pancreatic ductal neoplasms identified by gene expression profiling. Clin Cancer Res.

[B16] Oya M, Schulz WA (2000). Decreased expression of p57(KIP2)mRNA in human bladder cancer. British journal of cancer.

[B17] Hoffmann MJ, Florl AR, Seifert HH, Schulz WA (2005). Multiple mechanisms downregulate CDKN1C in human bladder cancer. International journal of cancer.

[B18] Kobatake T, Yano M, Toyooka S, Tsukuda K, Dote H, Kikuchi T, Toyota M, Ouchida M, Aoe M, Date H (2004). Aberrant methylation of p57KIP2 gene in lung and breast cancers and malignant mesotheliomas. Oncology reports.

[B19] Kondo M, Matsuoka S, Uchida K, Osada H, Nagatake M, Takagi K, Harper JW, Takahashi T, Elledge SJ, Takahashi T (1996). Selective maternal-allele loss in human lung cancers of the maternally expressed p57KIP2 gene at 11p15.5. Oncogene.

[B20] Pateras IS, Apostolopoulou K, Koutsami M, Evangelou K, Tsantoulis P, Liloglou T, Nikolaidis G, Sigala F, Kittas C, Field JK (2006). Downregulation of the KIP family members p27(KIP1) and p57(KIP2) by SKP2 and the role of methylation in p57(KIP2) inactivation in nonsmall cell lung cancer. International journal of cancer.

[B21] Chilosi M, Piazzola E, Lestani M, Benedetti A, Guasparri I, Granchelli G, Aldovini D, Leonardi E, Pizzolo G, Doglioni C (1998). Differential expression of p57KIP2, a maternally imprinted cdk inhibitor, in normal human placenta and gestational trophoblastic disease. Laboratory Investigation.

[B22] Thompson JS, Reese KJ, DeBaun MR, Perlman EJ, Feinberg AP (1996). Reduced expression of the cyclin-dependent kinase inhibitor gene p57KIP2 in Wilms' tumor. Cancer Research.

[B23] Hatada I, Ohashi H, Fukushima Y, Kaneko Y, Inoue M, Komoto Y, Okada A, Ohishi S, Nabetani A, Morisaki H (1996). An imprinted gene p57KIP2 is mutated in Beckwith-Wiedemann syndrome. Nature genetics.

[B24] Samuelsson MK, Pazirandeh A, Davani B, Okret S (1999). p57Kip2, a glucocorticoid-induced inhibitor of cell cycle progression in HeLa cells. Molecular endocrinology (Baltimore, Md).

[B25] Joaquin M, Watson RJ (2003). The cell cycle-regulated B-Myb transcription factor overcomes cyclin-dependent kinase inhibitory activity of p57(KIP2) by interacting with its cyclin-binding domain. The Journal of biological chemistry.

[B26] Scandura JM, Boccuni P, Massague J, Nimer SD (2004). Transforming growth factor beta-induced cell cycle arrest of human hematopoietic cells requires p57KIP2 up-regulation. Proceedings of the National Academy of Sciences of the United States of America.

[B27] Nijjar T, Wigington D, Garbe JC, Waha A, Stampfer MR, Yaswen P (1999). p57KIP2 expression and loss of heterozygosity during immortal conversion of cultured human mammary epithelial cells. Cancer Res.

[B28] Moggs JG, Murphy TC, Lim FL, Moore DJ, Stuckey R, Antrobus K, Kimber I, Orphanides G (2005). Anti-proliferative effect of estrogen in breast cancer cells that re-express ERalpha is mediated by aberrant regulation of cell cycle genes. Journal of molecular endocrinology.

[B29] Chisholm K, Bray BJ, Rosengren RJ (2004). Tamoxifen and epigallocatechin gallate are synergistically cytotoxic to MDA-MB-231 human breast cancer cells. Anti-cancer drugs.

[B30] Hsu S, Lewis JB, Borke JL, Singh B, Dickinson DP, Caughman GB, Athar M, Drake L, Aiken AC, Huynh CT (2001). Chemopreventive effects of green tea polyphenols correlate with reversible induction of p57 expression. Anticancer research.

[B31] Ravindranath MH, Saravanan TS, Monteclaro CC, Presser N, Ye X, Selvan SR, Brosman S (2006). Epicatechins Purified from Green Tea (Camellia sinensis) Differentially Suppress Growth of Gender-Dependent Human Cancer Cell Lines. Evid Based Complement Alternat Med.

[B32] Valcic S, Timmermann BN, Alberts DS, Wachter GA, Krutzsch M, Wymer J, Guillen JM (1996). Inhibitory effect of six green tea catechins and caffeine on the growth of four selected human tumor cell lines. Anti-cancer drugs.

[B33] Gudmundsson J, Barkardottir RB, Eiriksdottir G, Baldursson T, Arason A, Egilsson V, Ingvarsson S (1995). Loss of heterozygosity at chromosome 11 in breast cancer: association of prognostic factors with genetic alterations. British journal of cancer.

[B34] Karnik P, Paris M, Williams BRG, Casey G, Crowe J, Chen P (1998). Two distinct tumor suppressor loci within chromosome 11p15 implicated in breast cancer progression and metastatsis. Human Molecular Genetics.

[B35] Lichy JH, Zavar M, Tsai MM, O'Leary TJ, Taubenberger JK (1998). Loss of heterozygosity on chromosome 11p15 during histological progression in microdissected ductal carcinoma of the breast. The American journal of pathology.

[B36] Tokino T, Urano T, Furuhata T, Matsushima M, Miyatsu T, Sasaki S, Nakamura Y (1996). Characterization of the human p57KIP2 gene: alternative splicing, insertion/deletion polymorphisms in VNTR sequences in the coding region, and mutational analysis. Human Genetics.

[B37] Gene Expression Omnibus. http://www.ncbi.nlm.nih.gov.

[B38] Schlechter BL, Yang Q, Larson PS, Golubeva A, Blanchard RA, de las Morenas A, Rosenberg CL (2004). Quantitative DNA fingerprinting may distinguish new primary breast cancer from disease recurrence. J Clin Oncol.

[B39] Larson P, de Las Morenas A, Cerda S, Bennett S, Cupples L, Rosenberg C (2006). Quantitative analysis of allele imbalance supports atypical ductal hyperplasia lesions as direct breast cancer precursors. J Pathol.

[B40] Larson PS, Ungarelli RA, de Las Morenas A, Cupples LA, Rowlings K, Palmer JR, Rosenberg CL (2006). In utero exposure to diethylstilbestrol (DES) does not increase genomic instability in normal or neoplastic breast epithelium. Cancer.

[B41] Rosenberg CL, Finnemore EM, Larson PS, Nogueira CP, Delaney TL (2000). DNA alterations in tumor scrapes vs. biopsies of squamous-cell carcinomas of the head and neck. Int J Cancer.

[B42] King C, Guo N, Frampton GM, Gerry NP, Lenburg ME, Rosenberg CL (2005). Reliability and reproducibility of gene expression measurements using amplified RNA from laser-microdissected primary breast tissue with oligonucleotide arrays. J Mol Diagn.

[B43] Tricarico C, Pinzani P, Bianchi S, Paglierani M, Distante V, Pazzagli M, Bustin SA, Orlando C (2002). Quantitative real-time reverse transcription polymerase chain reaction: normalization to rRNA or single housekeeping genes is inappropriate for human tissue biopsies. Anal Biochem.

[B44] Kerangueven F, Noguchi T, Coulier F, Allione F, Wargniez V, Simony-Lafontaine J, Longy M, Jacquemier J, Sobol H, Eisinger F (1997). Genome-wide search for loss of heterozygosity shows extensive genetic diversity of human breast carcinomas. Cancer Res.

[B45] Osborne RJ, Hamshere MG (2000). A genome-wide map showing common regions of loss of heterozygosity/allelic imbalance in breast cancer. Cancer Res.

[B46] Shen CY, Yu JC, Lo YL, Kuo CH, Yue CT, Jou YS, Huang CS, Lung JC, Wu CW (2000). Genome-wide search for loss of heterozygosity using laser capture microdissected tissue of breast carcinoma: an implication for mutator phenotype and breast cancer pathogenesis. Cancer Res.

[B47] Lakhani S, Bissell M, Editors (2005). The role of myoepithelial cells in integration of form and function in the mammary gland. Journal of mammary gland biology and neoplasia.

[B48] Jones C, Mackay A, Grigoriadis A, Cossu A, Reis-Filho JS, Fulford L, Dexter T, Davies S, Bulmer K, Ford E (2004). Expression profiling of purified normal human luminal and myoepithelial breast cells: identification of novel prognostic markers for breast cancer. Cancer Res.

[B49] Figliola R, Maione R (2004). MyoD induces the expression of p57Kip2 in cells lacking p21Cip1/Waf1: overlapping and distinct functions of the two cdk inhibitors. Journal of cellular physiology.

